#  Avaliação da implantação de um projeto de telerregulação assistencial em uma capital brasileira 

**DOI:** 10.1590/0102-311XPT009623

**Published:** 2023-07-17

**Authors:** Michelle da Silva de Araújo, Ana Coelho de Albuquerque, Eronildo Felisberto, Isabella Samico, Átila Szczecinski Rodrigues

**Affiliations:** 1 Diretoria Executiva de Regulação em Saúde, Secretaria Municipal de Saúde de Recife, Recife, Brasil.; 2 Instituto de Medicina Integral Professor Fernando Figueira, Recife, Brasil.; 3 Agência para o Desenvolvimento da Atenção Primária à Saúde, Brasília, Brasil.

**Keywords:** Regulação e Fiscalização em Saúde, Acesso aos Serviços de Saúde, Telemedicina, Avaliação de Programas e Projetos de Saúde, Health Care Coordination and Monitoriang, Health Services Accessibility, Telemedicine, Program Evaluation, Regulación y Fiscalización en Salud, Accesibility a los Servicios de Salud, Telemedicina, Evaluación de Programas y Proyectos de Salud

## Abstract

Os objetivos foram avaliar o grau de implantação do projeto Regula+ Brasil e analisar em que medida as variações da implantação influenciam nos resultados observados no acesso a consultas especializadas em Recife, Pernambuco, Brasil. Trata-se de uma pesquisa avaliativa de análise de implantação. Foram elaborados o modelo lógico e a matriz de análise e julgamento com os indicadores para avaliação do grau de implantação e de resultado do projeto, os quais foram submetidos ao consenso de especialistas. A coleta de dados se deu por meio de questionário semiestruturado, aplicado com informantes-chave, e dados secundários extraídos dos documentos oficiais do projeto e do Sistema Nacional de Regulação (SISREG), referentes ao período de maio de 2020 a maio de 2021, os quais foram consolidados e comparados com valores definidos na matriz. O grau de implantação do projeto Regula+ Brasil em Recife foi considerado implantado (83,7%), bem como as dimensões Estrutura (81,7%) e Processo (84,6%). Entretanto, a maioria dos seus indicadores de efeito obtiveram desempenho insatisfatório. Quando confrontados, guardaram coerência com gargalos observados em alguns componentes e subcomponentes do projeto, como a atuação dos profissionais das unidades básicas de saúde (UBS), apontada como incipiente, principalmente no que diz respeito ao acompanhamento das solicitações devolvidas. Os resultados sugerem que qualquer intervenção em telessaúde requer, para sua devida implantação e para o alcance dos resultados esperados, adequação das equipes e dos processos de trabalho, práticas de educação permanente e processo contínuo de avaliação, ou então se configurará em nova burocratização e barreira de acesso.

## Abstract (en)

This study evaluates the degree of implementation (DI) of the Regulates+ Brazil project and analyzes to what extent the variations in implementation influence the results observed in access to specialized appointments in Recife, Pernambuco, Brazil. This is an evaluative research of implementation analysis. A logic model and an analysis and judgment matrix with indicators for evaluating the degree of implementation and the results of the Project were developed and submitted to expert consensus. The data collection was conducted via a semi-structured questionnaire applied to key informants and secondary data extracted from the official documents from Regulates+ Brazil and Brazilian National Regulation System (SISREG), referring to the period from May/2020 to May/2021, which were consolidated and compared with values defined in the matrix. The degree of implementation of the Regulates+ Brazil project in Recife was considered to be implemented (83.7%), as well as the Structure (81.7%) and Methods (84.6%) dimensions. However, most effect indicators underperformed, which, when confronted, were consistent with bottlenecks observed in some components and subcomponents of the Project, such as the performance of professionals in basic health units, which was indicated as incipient, especially regarding follow-up of returned requests. The results suggest that any intervention in Telehealth requires, for its proper implementation, the adequacy of teams and work processes, practices of Continuing Education, as well as a continuous evaluation process, to achieve the expected results, or else it will become another bureaucratization and barrier to access.

## Resumen

El objetivo fue evaluar el grado de implementación del proyecto Regula+ Brasil y analizar en qué medida las variaciones de la implementación influyen en los resultados observados en el acceso a consultas especializadas en Recife, Pernambuco, Brasil. Se trata de una investigación evaluativa del análisis de la implantación. Se elaboró un modelo lógico y una matriz de análisis y juicio con los indicadores para la evaluación de la grado de implementación y el resultado del Proyecto, los cuales se sometieron al consenso de especialistas. La recopilación de datos se realizó por medio de un cuestionario semiestructurado aplicado con informantes clave y datos secundarios extraídos de documentos oficiales del Regula+ Brasil y del Sistema Nacional de Regulación (SISREG), relativos al período de mayo de 2020 a mayo de 2021, los cuales fueron consolidados y comparados con valores definidos en el matriz. El grado de implementación del proyecto Regula+ Brasil en Recife fue considerado implementado (83,7%), así como las dimensiones Estructura (81,7%) y Proceso (84,6%). Sin embargo, la mayoría de sus indicadores de efecto obtuvieron un desempeño insatisfactorio, los cuales, al ser confrontados, fueron coherentes con los cuellos de botella observados en algunos componentes y subcomponentes del Proyecto, como el desempeño de los profesionales de las unidades básicas de salud, señalada como incipiente, sobre todo en lo que respecta al seguimiento de las solicitudes devueltas. Los resultados sugieren que toda intervención en Telesalud requiere, para su debida implantación, la adecuación de los equipos y de los procesos de trabajo, prácticas de Educación Permanente, así como un proceso continuo de evaluación, para alcanzar los resultados esperados, o entonces se configurará en una nueva burocratización y barrera de acceso.

## Introdução

A regulação em saúde busca alcançar os princípios da universalidade, integralidade e equidade no Sistema Único de Saúde (SUS) ^1^. No Brasil, a Política Nacional de Regulação organiza ações de regulação em saúde em três dimensões. Uma delas é a regulação do acesso à assistência, que se relaciona com organização, controle, gerenciamento e priorização do acesso e dos fluxos assistenciais no SUS, exercendo autoridade sanitária para a garantia do acesso, baseada em protocolos, classificação de risco e demais critérios de priorização ^2^.

A regulação do acesso à assistência, ou regulação assistencial, é um importante mecanismo de gestão entre oferta e demanda, canal de comunicação entre unidades de saúde, na busca por equidade ^1^^,^^3^^,^^4^. Entretanto, lacunas ainda existentes têm tornado a priorização oportunizada pela regulação assistencial insuficiente para satisfazer as necessidades de saúde da população ^5^. Esse cenário tem levado à formação de demandas reprimidas e, consequentemente, longas filas de espera ^1^, realidade que tem constituído um problema comum no sistema público de saúde do Brasil e do mundo ^6^.

A insuficiência de recursos financeiros e de serviços poderia ser apontada como o principal determinante para ocorrência dessas filas, porém fatores organizacionais e gerenciais, como a baixa resolubilidade da atenção primária à saúde (APS), encaminhamentos inapropriados, marcações desnecessárias para especialistas e problemas na contrarreferência têm sido igualmente citados como aspectos limitadores da regulação assistencial ^7^^,^^8^^,^^9^^,^^10^. A transposição dessa problemática tem exigido dos complexos reguladores a incorporação de novas estratégias e tecnologias ^11^, particularmente relacionadas a serviços de telessaúde. Sua inclusão no processo de regulação assistencial, denominada telerregulação, pode representar um grande passo na melhoria do acesso às consultas especializadas para que a regulação seja efetivamente uma valiosa ferramenta de gestão ^12^.

Em 2020, o Município de Recife, Pernambuco, Brasil, aderiu ao projeto Regula+ Brasil, visando, por meio da telerregulação, qualificar o processo de regulação assistencial e reduzir as filas de espera para algumas especialidades, bem como oferecer suporte no manejo clínico aos profissionais da APS por meio da telessaúde ^13^. Assim, os objetivos deste estudo foram avaliar o grau de implantação do projeto Regula+ Brasil no que diz respeito à estrutura e aos processos desenvolvidos e analisar em que medida as variações da implantação influenciam nos resultados observados na regulação assistencial de Recife.

## Método

Pesquisa avaliativa, de análise de implantação, que relaciona a influência da variação do grau de implantação de uma intervenção sobre os resultados observados ^14^.

A intervenção estudada foi o projeto Regula+ Brasil, desenvolvido no âmbito do Programa de Apoio ao Desenvolvimento Institucional do SUS (PROADI-SUS). O PROADI-SUS é uma aliança entre Entidades de Saúde de Reconhecida Excelência (ESRE) - hospitais de referência no Brasil (Hospital Israelita Albert Einstein, HCor [Associação Beneficente Síria], Hospital Moinhos de Vento, Hospital Alemão Oswaldo Cruz e Hospital Sírio-Libanês) - e o Ministério da Saúde, com os propósitos de apoiar e aprimorar o SUS por meio de projetos de capacitação de recursos humanos, pesquisa, avaliação e incorporação de tecnologias, gestão e assistência especializada.

O projeto atuou em duas frentes: a teleconsultoria, para apoiar os médicos das unidades básicas de saúde (UBS), e a telerregulação, para orientar a regulação das filas para consultas na atenção secundária.

Selecionou-se Recife como local de estudo, capital do Estado de Pernambuco, município considerado como o segundo maior polo médico do Brasil, com 2.116 estabelecimentos de saúde, 1.820 da rede privada e 296 da pública, 59 tipos de serviços especializados e mais de oito mil leitos hospitalares ^15^. O acesso à rede especializada ocorre por meio de encaminhamentos realizados pelos profissionais da APS (médicos e odontólogos), mediante o Sistema Nacional de Regulação (SISREG), nas unidades de saúde de referência dos usuários.

Este estudo toma como referência o período de maio de 2020 a maio de 2021, meses estabelecidos no relatório final do projeto.

**Figura 1:**
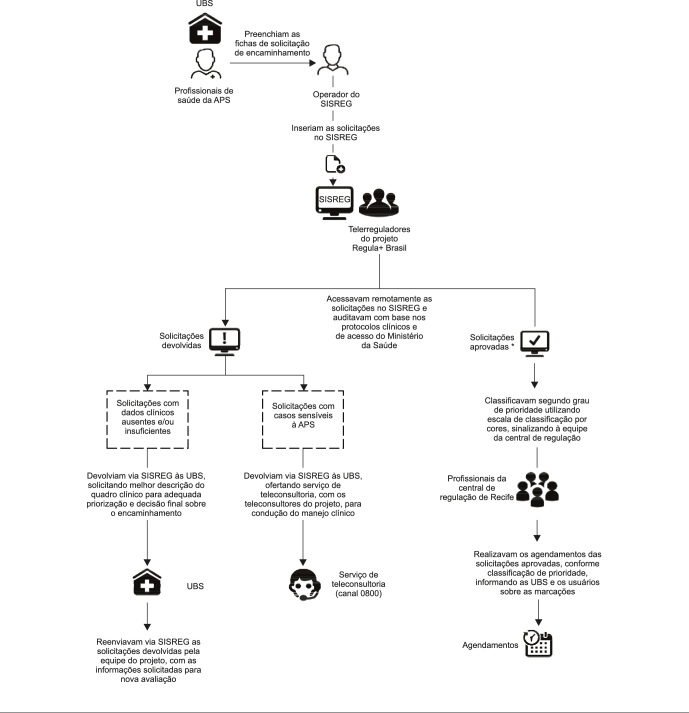
Fluxo das atividades do projeto Regula+ Brasil. Recife, Pernambuco, Brasil.

APS: atenção primária à saúde; SISREG: Sistema Nacional de Regulação; UBS: unidades básicas de saúde. Fonte: elaboração dos autores e Gardez et al. ^16^. * Nos casos aprovados com perfil para atendimento remoto, a equipe do projeto tentava entrar em contato com os usuários para agendar o serviço de teleconsulta com um dos especialistas do projeto (excepcionalmente durante a pandemia).

As atividades regulatórias do projeto iniciaram-se após análise prévia das filas e definição das especialidades que seriam reguladas (Figura 1) ^16^. Foram definidas as especialidades de cardiologia adulto (geral), endocrinologia adulto (geral e pacientes diabéticos), neurologia (adulto), reumatologia (adulto), traumato-ortopedia (adulto) e, posteriormente, psiquiatria (adulto).

### Etapas do estudo avaliativo

O estudo avaliativo foi composto por cinco etapas: (1) elaboração e validação do modelo lógico; (2) elaboração e validação da matriz de análise e julgamento; (3) classificação do grau de implantação; (4) análise dos indicadores de efeitos; e (5) análise da influência do grau de implantação sobre os efeitos observados.

**Figura 2:**
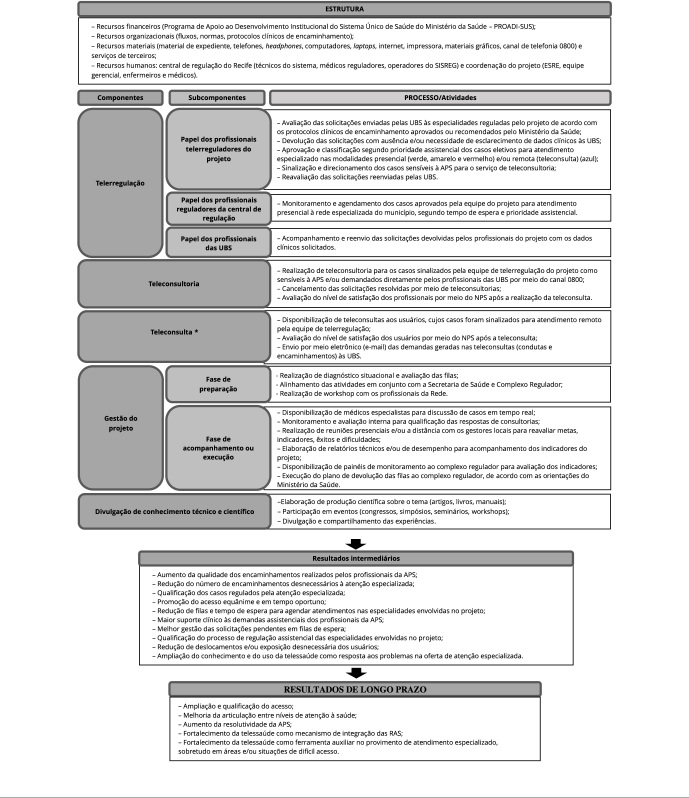
Modelo lógico final do projeto Regula+ Brasil. Recife, Pernambuco, Brasil, 2022.

APS: atenção primária à saúde; ESRE: entidades de reconhecida excelência; NPS: *net promoter score*; RAS: redes de atenção à saúde; SISREG: Sistema Nacional de Regulação; UBS: unidades básicas de saúde. * Componente oferecido em caráter emergencial devido à pandemia de COVID-19, não fazendo parte do escopo inicial do projeto.

Para descrever a intervenção, elaborou-se uma versão inicial do modelo lógico do Regula+ Brasil, utilizando como referência documentos oficiais e legislações, em que foram definidos os componentes das dimensões estrutura e processo ^17^, bem como os resultados esperados. Esse modelo foi submetido a especialistas ligados ao projeto no Ministério da Saúde (n = 2), no hospital de excelência (n = 3), na central de regulação da Secretaria Municipal de Saúde (SMS) de Recife (n = 4) e à avaliação em saúde (n = 3), perfazendo um total de 12 profissionais, que puderam acrescentar ou suprimir informações se julgassem necessário. O processo aconteceu em duas rodadas e o modelo foi considerado finalizado quando os especialistas não tiveram mais alterações a fazer (Figura 2).

Com base no modelo lógico final, uma matriz de análise e julgamento foi elaborada e submetida à consulta e validação dos especialistas supracitados, sendo concluída em duas rodadas, composta pelos componentes (e subcomponentes), pelas respectivas pontuações e pelas fontes de dados para cada indicador (Quadro 1).

**Quadro 1:** Matriz de análise e julgamento final do projeto Regula+ Brasil (estrutura, processo e resultado). Recife, Pernambuco, Brasil, 2022.

DIMENSÃO ESTRUTURA					
Itens	Indicador	Padrão	Fonte de verificação	Descrição do valor ou ponto de corte	
Recursos financeiros (PROADI-SUS) Recursos organizacionais (fluxos, normas, protocolos de encaminhamento/acesso) Recursos materiais (material de expediente, telefones, *headphones*, computadores, *laptops*, internet, impressora, materiais gráficos, canal de telefonia 0800) e serviços de terceiros Recursos humanos: Central de regulação do Recife: técnicos do sistema, médicos reguladores, operadores do SISREG Coordenação do projeto (hospital de excelência): equipe gerencial, enfermeiros e médicos	Existência de legislação atualizada no último ano para rotina ou situações de emergência (surtos, epidemias, desastres e outros eventos inusitados)	Sim	EIC	Sim = 2,0	
				Não = 0,0	
	Existência de fluxos e protocolos de encaminhamento/acesso (locais ou nacionais)	Sim. Suficiente	EIC	Sim. Suficiente = 2,0	
				Sim. Insuficiente = 1,0	
				Não = 0,0	
	Disponibilidade de recursos materiais (material de expediente, telefones, *headphones*, computadores, *laptops*, internet, impressora etc.) e de serviços de terceiros suficientes para o desenvolvimento do projeto	Sim. Suficiente	EIC	Sim. Suficiente = 2,0	
				Sim. Insuficiente = 1,0	
				Não = 0,0	
	Existência de um complexo regulador que utilize um sistema para agendar atendimentos (próprio ou nacional) e que tenha filas de espera	Sim	EIC	Sim = 2,0	
				Não = 0,0	
	Existência de técnicos em regulação	Sim. Suficiente	EIC	Sim. Suficiente = 2,0	
				Sim. Insuficiente = 1,0	
				Não = 0,0	
	Existência de médicos reguladores no complexo regulador	Sim. Suficiente	EIC	Sim. Suficiente = 2,0	
				Sim. Insuficiente = 1,0	
				Não = 0,0	
	Existência de operadores do SISREG para administração das demandas geradas no sistema nas UBS	Sim. Suficiente	EIC	Sim. Suficiente = 2,0	
				Sim. Insuficiente = 1,0	
				Não = 0,0	
	Existência de equipe gerencial para o desenvolvimento do projeto nos hospitais de excelência	Sim. Suficiente	EIC	Sim. Suficiente = 2,0	
				Sim. Insuficiente = 1,0	
				Não = 0,0	
	Existência de médicos para o desenvolvimento do projeto nos hospitais de excelência	Sim. Suficiente	EIC	Sim. Suficiente = 2,0	
				Sim. Insuficiente = 1,0	
				Não = 0,0	
	Existência de enfermeiros para o desenvolvimento do projeto nos hospitais de excelência	Sim. Suficiente	EIC	Sim. Suficiente = 2,0	
				Sim. Insuficiente = 1,0	
				Não = 0,0	
TOTAL GRAU DE IMPLANTAÇÃO (ESTRUTURA)				20,0 (pontuação máxima)	
DIMENSÃO PROCESSO					
Componente: TELERREGULAÇÃO					
Subcomponentes	Atividades	Indicador	Padrão	Fonte de verificação	Descrição do valor ou ponto de corte
Papel dos profissionais telerreguladores do projeto	Avaliação das solicitações enviadas pelas UBS às especialidades reguladas pelo projeto de acordo com os protocolos de encaminhamento aprovados pelo Ministério da Saúde	Número de solicitações avaliadas	50.000 solicitações avaliadas	Relatórios de acompanhamento/banco de dados do projeto/*dashboards*	≥ 50.000 = 2,0
					33.333-50.000 = 1,0
					< 3.333 = 0,0
	Devolução das solicitações com ausência e/ou necessidade de esclarecimento de dados clínicos às UBS	Realização de rotina de devolução das solicitações com ausência e/ou necessidade de esclarecimento de dados clínicos às UBS	Sim, sempre que necessário	EIC	Sim, sempre que necessário = 2,0
					Sim, na maioria das vezes = 1,5
					Sim, às vezes = 1,0
					Sim, raramente = 0,5
					Nunca = 0,0
	Aprovação e classificação segundo prioridade assistencial dos casos eletivos para atendimento especializado nas modalidades presencial (verde, amarelo e vermelho) e/ou remota (teleconsulta/teleatendimento) (azul)	Realização de processo de avaliação e classificação segundo grau de prioridade assistencial das solicitações pelos profissionais do projeto	Sim, sempre que necessário	EIC	Sim, sempre que necessário = 2,0
					Sim, na maioria das vezes = 1,5
					Sim, às vezes = 1,0
					Sim, raramente = 0,5
					Nunca = 0,0
	Sinalização e direcionamento dos casos sensíveis à APS para o serviço de teleconsultoria	Realização de processo de sinalização e direcionamento dos casos sensíveis à APS para o serviço de teleconsultoria identificados das filas	Sim, sempre que necessário	EIC	Sim, sempre que necessário = 2,0
					Sim, na maioria das vezes = 1,5
					Sim, às vezes = 1,0
					Sim, raramente = 0,5
					Nunca = 0,0
	Reavaliação das solicitações reenviadas pelas UBS	Percentual de solicitações reavaliadas após devolução para as UBS	10%-25% das solicitações reavaliadas após devolução para as UBS	Relatórios de acompanhamento/banco de dados do projeto/*dashboards*	10%-25% ou mais = 2,0
					5%-10% = 1,0
					< 5% = 0,0
Papel dos profissionais reguladores da central de regulação	Monitoramento e regulação dos casos aprovados pela equipe do projeto para atendimento presencial à rede especializada do município, segundo tempo de espera e prioridade assistencial	Existência de processo de monitoramento e regulação dos casos aprovados pela equipe do projeto	Sim, constantemente	EIC	Sim, constantemente = 2,0
					Sim, mas não constantemente = 1,0
					Não = 0,0
		Percentual de agendamento dos casos aprovados pela equipe do projeto	≥ 75% das solicitações aprovadas no período	Banco de dados do SISREG	≥ 75% = 2,0
					50%-75% = 1,0
					25%-50% = 0,5
					< 25% = 0,0
Papel dos profissionais das UBS	Acompanhamento e reenvio das solicitações devolvidas pelos profissionais do projeto com os dados clínicos solicitados	Existência de rotina de acompanhamento e reenvio das solicitações devolvidas pelos profissionais do projeto com os dados clínicos solicitados	Sim, sempre que necessário	EIC	Sim, sempre que necessário = 2,0
					Sim, na maioria das vezes = 1,5
					Sim, às vezes = 1,0
					Sim, raramente = 0,5
					Nunca = 0,0
Componente: TELECONSULTORIA					
Subcomponentes	Atividades	Indicador	Padrão	Fonte de verificação	Descrição do valor ou ponto de corte
	Realização de teleconsultoria para os casos sinalizados pela equipe de telerregulação do projeto como sensíveis à APS e/ou demandados diretamente pelos profissionais das UBS por meio do canal 0800	Oferta de teleconsultorias aos profissionais solicitantes das UBS	Sim. Suficiente	EIC	Sim. Suficiente = 2,0
					Sim. Insuficiente = 1,0
					Não = 0,0
		Grau de adesão dos profissionais solicitantes às teleconsultorias	Alto ou muito alto	EIC	Muito alto = 2,0
					Alto = 1,5
					Baixo = 1,0
					Muito baixo = 0,5
					Inexistente = 0,0
		Percentual de solicitações reavaliadas após devolução para APS, por consultoria em tempo real	3%-15%	Relatórios de acompanhamento/banco de dados do projeto/*dashboards*	> 3%-15% ou mais = 2,0
					1%-3% = 1,0
					0% = 0,0
	Cancelamento das solicitações resolvidas nas teleconsultorias informando às UBS o motivo	Realização de cancelamento de solicitações resolvidas nas teleconsultorias	Sim, sempre que necessário	EIC	Sim, sempre que necessário = 2,0
					Sim, na maioria das vezes = 1,5
					Sim, às vezes = 1,0
					Sim, raramente = 0,5
					Nunca = 0,0
	Avaliação do nível de satisfação dos profissionais por meio do NPS após a realização da teleconsulta	Realização de avaliação do nível de satisfação dos profissionais após as teleconsultas	Sim	EIC	Sim = 1,0
					Não = 0,0
Componente: TELECONSULTA					
Subcomponentes	Atividades	Indicador	Padrão	Fonte de verificação	Descrição do valor ou ponto de corte
	Disponibilização de teleconsultas aos usuários, cujos casos foram sinalizados para atendimento remoto pela equipe de telerregulação	Realização de teleconsultas aos usuários dos casos sinalizados para atendimento remoto	Sim, sempre que sinalizado para atendimento remoto	EIC	Sim, sempre que sinalizado = 2,0
					Sim, na maioria das vezes = 1,5
					Sim, às vezes = 1,0
					Sim, raramente = 0,5
					Nunca = 0,0
	Avaliação do nível de satisfação dos usuários por meio do NPS após a teleconsulta	Realização de avaliação do nível de satisfação dos usuários após as teleconsultas	Sim	EIC	Sim = 1,0 Não = 0,0
	Envio por meio eletrônico (e-mail) das demandas geradas nas teleconsultas (condutas e encaminhamentos) às UBS	Existência de fluxo de comunicação das demandas geradas nas teleconsultas	Sim	EIC	Sim = 1,0
					Não = 0,0
Componente: GESTÃO DO PROJETO					
Subcomponentes	Atividades	Indicador	Padrão	Fonte de verificação	Descrição do valor ou ponto de corte
Fase de preparação	Realização de diagnóstico situacional e avaliação das filas	Existência de diagnóstico situacional e avaliação das filas	Sim.	EIC	Sim = 1,0
					Não = 0,0
	Realização de reuniões de alinhamento das atividades em conjunto com a secretaria de saúde e com o complexo regulador	Existência de reuniões de alinhamento entre ESRE, SMS e o complexo regulador	Sim	EIC	Sim = 1,0
					Não = 0,0
	Realização de workshop com os profissionais da rede	Existência de workshop com os profissionais da rede	Sim	EIC	Sim = 1,0
					Não = 0,0
	Definição de indicadores de monitoramento e metas estabelecidas pela equipe do projeto e pelo complexo regulador	Existência de indicadores de monitoramento e metas estabelecidas pela equipe do projeto e pelo complexo regulador	Sim	EIC	Sim = 1,0
					Não = 0,0
Fase de acompanhamento ou execução	Monitoramento e avaliação interna para qualificação das respostas das consultorias	Existência de monitoramento e avaliação das respostas das consultorias	Sim	EIC	Sim = 1,0
					Não = 0,0
	Realização de reuniões presenciais e/ou a distância com os gestores locais para reavaliar metas, indicadores, êxitos e dificuldades	Existência de reuniões presenciais e/ou a distância com os gestores locais para avaliação de metas e indicadores durante a execução do projeto	Sim	EIC	Sim = 1,0
					Não = 0,0
	Elaboração de relatórios técnicos e/ou de desempenho para acompanhamento dos indicadores do projeto	Elaboração de relatórios técnicos e/ou de desempenho para acompanhamento dos indicadores do projeto	Sim	EIC	Sim = 1,0
					Não = 0,0
	Disponibilização de painéis de monitoramento ao complexo regulador para avaliação dos indicadores	Atualização dos painéis de monitoramento compartilhados com o complexo regulador para avaliação dos indicadores, mensalmente	Sim	EIC	Sim = 1,0
					Não = 0,0
	Estabelecimento de canal de comunicação entre a equipe do projeto, a central de regulação e a UBS	Existência de canal de comunicação a entre equipe do projeto, a central de regulação e a UBS	Sim	EIC	Sim = 1,0
					Não = 0,0
	Execução do plano de devolução das filas ao complexo regulador, de acordo com as orientações do Ministério da Saúde	Execução do plano de devolução das filas ao complexo regulador, de acordo com as orientações do Ministério da Saúde	Sim	EIC	Sim = 1,0
					Não = 0,0
Componente: DIVULGAÇÃO DE CONHECIMENTO TÉCNICO E CIENTÍFICO					
Subcomponentes	Atividades	Indicador	Padrão	Fonte de verificação	Descrição do valor ou ponto de corte
	Elaboração de produção científica sobre o tema (artigos, livros, manuais)	Existência de produção científica sobre o tema durante o período de execução do projeto	Sim	EIC	Sim = 1,0
					Não = 0,0
	Participação em eventos (congressos, simpósios, seminários, workshops)	Participação em eventos (congressos, simpósios, seminários, workshops) durante o período de execução do projeto	Sim	EIC	Sim = 1,0
					Não = 0,0
	Divulgação e compartilhamento das experiências	Realização de divulgação e compartilhamento das experiências durante o período de execução do projeto	Sim	EIC	Sim = 1,0
					Não = 0,0
Total grau de implantação (Processo)				43,0 (pontuação máxima)	
Grau de implantação total (Estrutura e Processo)				63,0 (pontuação máxima)	
DIMENSÃO RESULTADO					
Resultados intermediários	Indicadores	Metas		Fontes de verificação	
Aumento da qualidade dos encaminhamentos realizados pelos profissionais da APS	Solicitações aprovadas na primeira avaliação (%) - total e por especialidade	Aumento de duas vezes ou atingir 40%		Relatórios de acompanhamento/banco de dados do projeto/*dashboards*	
Qualificação dos casos regulados para atenção especializada	Agendamentos de casos regulados prioritários (vermelho e amarelo) para atendimento especializado (%)	Incremento de 10% em relação ao ano anterior		Banco de dados do SISREG	
Qualificação do processo de regulação assistencial das especialidades envolvidas no projeto	Redução do tempo médio em dias de espera para autorização dos casos enviados às especialidades (%)	Redução de 25% ou mais em relação ao ano anterior		Banco de dados do SISREG	
Redução do número de encaminhamentos desnecessários à atenção especializada	Redução de novos encaminhamentos nas filas de espera (%) - total e por especialidade	Redução de no mínimo 15%		Relatórios de acompanhamento/banco de dados do projeto/*dashboards*	
Promoção do acesso equânime e em tempo oportuno	Redução do tempo médio em dias de espera nos casos regulados em prioridade alta (%) - total e por especialidade	Redução de no mínimo 25%		Relatórios de acompanhamento/banco de dados do projeto/*dashboards*	
	Redução do tempo médio em dias de espera nos casos regulados em prioridade média e baixa (%) - total e por especialidade	Redução de no mínimo 25%		Relatórios de acompanhamento/banco de dados do projeto/*dashboards*	
Redução de filas e tempo de espera para agendar atendimentos nas especialidades envolvidas no projeto	Redução no número de encaminhamentos em fila de espera (%) - total e por especialidade	Redução no mínimo de 40%		Relatórios de acompanhamento/banco de dados do projeto/*dashboards*	
Melhor gestão das solicitações pendentes em filas de espera					
Maior suporte clínico às demandas assistenciais dos profissionais da APS	Nível de satisfação dos profissionais atendidos nas teleconsultas	> 85 NPS		Relatórios de acompanhamento/banco de dados do projeto/*dashboards*	
Ampliação do conhecimento e do uso da telessaúde como resposta aos problemas na oferta de atenção especializada	Nível de satisfação dos usuários atendidos nas teleconsultas	> 85 NPS		Relatórios de acompanhamento/banco de dados do projeto/*dashboards*	

APS: atenção primária à saúde; EIC: entrevista com informantes-chave; ESRE: entidades de reconhecida

excelência; NPS: *net promoter score*; PROADI-SUS: Programa de Apoio ao Desenvolvimento Institucional do SUS; SISREG: Sistema Nacional de Regulação; SMS: Secretaria Municipal de Saúde; UBS: unidades básicas de saúde.

Fonte: elaboração própria.

A seleção dos indicadores considerou como critérios de inclusão a relevância, a disponibilidade e a facilidade de coleta de dados, atribuindo-se para cada um o padrão, a fonte de verificação e os pontos de corte distribuídos de acordo com a importância e o peso de seus componentes. Para definir os padrões, utilizaram-se aqueles estabelecidos nos documentos oficiais do próprio projeto. No caso dos indicadores propostos pelos pesquisadores, os padrões foram definidos em consonância com a realidade do município.

A coleta de dados foi feita mediante a aplicação de questionário semiestruturado a sete informantes-chave ligados ao projeto no hospital de excelência e no Ministério da Saúde e a gestores e médicos reguladores da central de regulação da SMS de Recife. Além das perguntas específicas, elaboradas a partir da matriz, foram elencadas questões relacionadas ao contexto de implantação do projeto, as quais puderam contribuir para o entendimento acerca dos achados sobre o grau de implantação. As respostas foram sistematizadas em uma matriz de análise, sendo identificados padrões ou temas que permitissem interpretar os dados, digitados e consolidados nos programas Microsoft Office Excel 2016 e Microsoft Office Word 2016 (https://products.office.com/).

Para obter o grau de implantação de estrutura e processo, foram utilizadas as pontuações finais alcançadas pelos seus respectivos indicadores, calculadas a partir da média ponderada resultante da soma de pontos obtidos pelo total de respostas. Os cálculos dos grau de implantação de cada dimensão, componente e subcomponente foram auferidos por meio dos valores percentuais resultantes do somatório dos pontos obtidos pelas pontuações máximas esperadas. O grau de implantação total foi estimado a partir do valor percentual do total geral de pontos obtidos pelo total máximo esperado para o projeto. Ao final, adotou-se a seguinte classificação: implantado (≥ 75%), parcialmente implantado (50%-75%), incipiente (25%-50%) e não implantado (< 25%).

Para analisar os efeitos (resultados), foram considerados nove indicadores de resultado e suas metas, de acordo com o modelo lógico, bem como aqueles já previstos no escopo do projeto, obedecendo a critérios de validade, relevância e disponibilidade de informação (Quadro 1). Utilizaram-se dados secundários extraídos de documentos oficiais - notas técnicas, relatórios de monitoramento e acompanhamento (*dashboards*) - e dos bancos de dados de fila e agendamento do SISREG. Os indicadores de resultado foram submetidos à análise dos especialistas durante as rodadas de consulta.

Este estudo foi aprovado pelo Comitê de Ética em Pesquisa em Seres Humanos do Instituto de Medicina Integral Professor Fernando Figueira (IMIP, sob o registro nº 52161121.5.0000.5201), e seguiu as recomendações da Resolução nº 466/2012, do Conselho Nacional de Saúde e complementares.

## Resultados

### Grau de implantação total do projeto Regula+ Brasil e suas dimensões - estrutura e processo

O grau de implantação total do projeto Regula+ Brasil no Município do Recife foi considerado implantado (83,7%). Quanto às dimensões, a estrutura e o processo também foram consideradas implantados (81,7% e 84,6%, respectivamente) (Tabela 1).

**Tabela 1:** Grau de implantação (dimensões estrutura e processo) do projeto Regula+ Brasil. Recife, Pernambuco, Brasil, 2022.

	Descrição do valor ou ponto de corte	Total das respostas	Soma dos pontos	Média final dos pontos
DIMENSÃO ESTRUTURA				
Indicador				
Existência de legislação atualizada no último ano para rotina ou situações de emergência (surtos, epidemias, desastres e outros eventos inusitados)	Sim = 2,0	4	8	2,00
	Não = 0,0	0	0	
Existência de fluxos e protocolos de encaminhamento/acesso (locais ou nacionais)	Sim. Suficiente = 2,0	1	2	1,10
	Sim. Insuficiente = 1,0	6	6	
	Não = 0,0	0	0	
Disponibilidade de recursos materiais (material de expediente, telefones, *headphones*, computadores, *laptops*, internet, impressora etc.) e de serviços de terceiros suficientes para o desenvolvimento do projeto	Sim. Suficiente = 2,0	2	4	2,00
	Sim. Insuficiente = 1,0	0	0	
	Não = 0,0	0	0	
Existência de um complexo regulador que utilize um sistema para agendar atendimentos (próprio ou nacional) e que tenha filas de espera	Sim = 2,0	6	12	2,00
	Não = 0,0	0	0	
Existência de técnicos em regulação	Sim. Suficiente = 2,0	1	2	1,20
	Sim. Insuficiente = 1,0	4	4	
	Não = 0,0	0	0	
Existência de médicos reguladores no complexo regulador	Sim. Suficiente = 2,0	1	2	1,20
	Sim. Insuficiente = 1,0	4	4	
	Não = 0,0	0	0	
Existência de operadores do SISREG para administração das demandas geradas no sistema nas UBS	Sim. Suficiente = 2,0	0	0	0,80
	Sim. Insuficiente = 1,0	4	4	
	Não = 0,0	1	0	
Existência de equipe gerencial para o desenvolvimento do projeto nos hospitais de excelência	Sim. Suficiente = 2,0	2	4	2,00
	Sim. Insuficiente = 1,0	0	0	
	Não = 0,0	0	0	
Existência de médicos para o desenvolvimento do projeto nos hospitais de excelência	Sim. Suficiente = 2,0	2	4	2,00
	Sim. Insuficiente = 1,0	0	0	
	Não = 0,0	0	0	
Existência de enfermeiros para o desenvolvimento do projeto nos hospitais de excelência	Sim. Suficiente = 2,0	2	4	2,00
	Sim. Insuficiente = 1,0	0	0	
	Não = 0,0	0	0	
Total pontuação (Estrutura)	20,00 (pontuação máxima)	-	-	16,30 (pontuação obtida)
Grau de implantação (Estrutura)	81,70% (implantado)			
DIMENSÃO PROCESSO				
Componente/Subcomponente/Indicador				
Telerregulação				
Papel dos profissionais telerreguladores do projeto				
Número de solicitações avaliadas	≥ 50.000 = 2,0	93.210	2	2,00
	33.333-50.000 = 1,0			
	< 33.333 = 0,0			
			
Realização de rotina de devolução das solicitações com ausência e/ou necessidade de esclarecimento de dados clínicos às UBS	Sim, sempre que necessário = 2,0	7	14	2,00
	Sim, na maioria das vezes = 1,5	0	0	
	Sim, às vezes = 1,0	0	0	
	Sim, raramente = 0,5	0	0	
	Nunca = 0,0	0	0	
Realização de processo de avaliação e classificação segundo grau de prioridade assistencial das solicitações pelos profissionais do projeto	Sim, sempre que necessário = 2,0	7	14	2,00
	Sim, na maioria das vezes = 1,5	0	0	
	Sim, às vezes = 1,0	0	0	
	Sim, raramente = 0,5	0	0	
	Nunca = 0,0	0	0	
Realização de processo de sinalização e direcionamento dos casos sensíveis à APS para o serviço de teleconsultoria identificados das filas	Sim, sempre que necessário = 2,0	6	12	2,00
	Sim, na maioria das vezes = 1,5	0	0	
	Sim, às vezes = 1,0	0	0	
	Sim, raramente = 0,5	0	0	
	Nunca = 0,0	0	0	
Percentual de solicitações reavaliadas após devolução para as UBS	10%-25% ou mais = 2,0	15%	2	2,00
	5%-10% = 1,0			
	< 5% = 0,0			
Grau de implantação subcomponente				100,0%
Papel dos profissionais reguladores da central de regulação				
Existência de processo de monitoramento e regulação dos casos aprovados pela equipe do projeto	Sim, constantemente = 2,0	2	4	0,86
	Sim, mas não constantemente = 1,0	2	2	
	Não = 0,0	3	0	
Percentual de agendamento dos casos aprovados pela equipe do projeto	≥ 75% = 2,0	92%	2	2,00
	50%-75% = 1,0			
	25%-50% = 0,5			
Grau de implantação subcomponente				71,40%
Papel dos profissionais das UBS				
Existência de rotina de acompanhamento e reenvio das solicitações devolvidas pelos profissionais do projeto com os dados clínicos solicitados.	Sim, sempre que necessário = 2,0	0	0	0,64
	Sim, na maioria das vezes = 1,5	0	0	
	Sim, às vezes = 1,0	2	2	
	Sim, raramente = 0,5	5	2,5	
	Nunca = 0,0	0	0	
Grau de implantação do subcomponente				32,10%
Grau de implantação do componente (Telerregulação)				84,40%
Teleconsultoria				
Oferta de teleconsultorias aos profissionais solicitantes das UBS	Sim. Suficiente = 2,0	4	8	2,00
	Sim. Insuficiente = 1,0	0	0	
	Não = 0,0	0	0	
Grau de adesão dos profissionais solicitantes às teleconsultorias	Muito alto = 2,0	0	0	0,70
	Alto = 1,5	0	0	
	Baixo = 1,0	2	2	
	Muito baixo = 0,5	3	1,5	
	Inexistente = 0,0	0	0	
Percentual de solicitações reavaliadas após devolução para APS, por consultoria, em tempo real	> 3%-15% ou mais = 2,0	1%	1	1,00
	1%-3% = 1,0			
	0% = 0,0			
Realização de cancelamento de solicitações resolvidas nas teleconsultorias	Sim, sempre que necessário = 2,0	2	4	2,00
	Sim, na maioria das vezes = 1,5	0	0	
	Sim, às vezes = 1,0	0	0	
	Sim, raramente = 0,5	0	0	
	Nunca = 0,0	0	0	
Realização de avaliação do nível de satisfação dos profissionais após as teleconsultas	Sim = 1,0	2	2	1,00
	Não = 0,0	0	0	
Grau de implantação do componente (Teleconsultoria)				74,40%
Teleconsultas				
Realização de teleconsultas aos usuários dos casos sinalizados para atendimento remoto	Sim, sempre que sinalizado = 2,0	5	10	1,90
	Sim, na maioria das vezes = 1,5	1	1,5	
	Sim, às vezes = 1,0	0	0	
	Sim, raramente = 0,5	0	0	
	Nunca = 0,0	0	0	
Realização de avaliação do nível de satisfação dos usuários após as teleconsultas	Sim = 1,0	2	2	1,00
	Não = 0,0	0	0	
Existência de fluxo de comunicação das demandas geradas nas teleconsultas	Sim = 1,0	3	3	1,00
	Não = 0,0	0	0	
Grau de implantação do componente (Teleconsultas)				97,90%
Gestão do projeto				
Fase de preparação				
Existência de diagnóstico situacional e avaliação das filas	Sim = 1,0	3	3	1,00
	Não = 0,0	0	0	
Existência de reuniões de alinhamento entre a ESRE, a SMS e o complexo regulador	Sim = 1,0	5	5	1,00
	Não = 0,0	0	0	
Existência de workshop com os profissionais da rede	Sim = 1,0	1	1	0,20
	Não = 0,0	4	0	
Existência de indicadores de monitoramento e metas estabelecidas pela equipe do projeto e pelo complexo regulador	Sim = 1,0	3	3	1,00
	Não = 0,0	0	0	
Grau de implantação do subcomponente				80,00%
Fase de acompanhamento ou execução				
Existência de monitoramento e avaliação das respostas das consultorias	Sim = 1,0	2	2	1,00
	Não = 0,0	0	0	
Existência de reuniões presenciais e/ou a distância com os gestores locais para avaliação de metas e indicadores durante a execução do projeto	Sim = 1,0	6	6	1,00
	Não = 0,0	0	0	
Elaboração de relatórios técnicos e/ou de desempenho para acompanhamento dos indicadores do projeto	Sim = 1,0	3	3	0,80
	Não = 0,0	1	0	
Atualização dos painéis de monitoramento compartilhados com o complexo regulador para avaliação dos indicadores, mensalmente	Sim = 1,0	2	2	0,50
	Não = 0,0	2	0	
Existência de canal de comunicação entre a equipe do projeto, a central de regulação e a UBS	Sim = 1,0	6	6	1,00
	Não = 0,0	0	0	
Execução do plano de devolução das filas ao complexo regulador de acordo com as orientações do Ministério da Saúde	Sim = 2,0	6	12	2,00
	Não = 0,0	0	0	
Grau de implantação do subcomponente				89,30%
Grau de implantação do componente (Gestão de projetos)				85,90%
Divulgação de conhecimento técnico e científico				
Existência de produção científica sobre o tema durante o período de execução do projeto	Sim = 1,0	1	1	1,00
	Não = 0,0	0	0	
Participação em eventos (congressos, simpósios, seminários, workshops) durante o período de execução do projeto	Sim = 1,0	1	1	1,00
	Não = 0,0	0	0	
Realização de divulgação e compartilhamento das experiências durante o período de execução do projeto	Sim = 1,0	4	4	0,80
	Não = 0,0	1	0	
Grau de implantação do componente (Divulgação de conhecimento técnico e científico)				93,30%
Total de pontuação (Processo)	43,00 (pontuação máxima)	-	-	36,40 (pontuação obtida)
Grau de implantação (Processo)	84,60% (implantado)			
Grau de implantação total	83,70% (implantado)			

APS: atenção primária à saúde; EIC: entrevista com informantes-chave; ESRE: entidades de reconhecida excelência; NPS: *net promoter score*; SISREG: Sistema Nacional de Regulação; SMS: Secretaria Municipal de Saúde; UBS: unidades básicas de saúde.

Fonte: elaboração própria.

Nota: classificação do grau de implantação: implantado (≥ 75%); parcialmente implantado (50%-75%); incipiente (25% a 50%); e não implantado (< 25%).

Alguns indicadores de estrutura tiveram resultados negativos, tais como “existência de fluxos e protocolos de encaminhamento/acesso”, “existência de técnicos em regulação”, “existência de médicos reguladores no complexo regulador” e “existência de operadores do SISREG para administração das demandas geradas no sistema nas UBS”. Os informantes-chave justificaram que a incoerência dos protocolos clínicos e de acesso utilizados pela equipe do projeto em relação à realidade do município e o baixo quantitativo de profissionais, tanto na central de regulação quanto nas UBS (operadores do SISREG), foram fatores que influenciaram nesse resultado.

Na dimensão processo, a maioria dos componentes foi considerada implantada, com destaque para telerregulação (84,4%), teleconsulta (97,9%), gestão do projeto (85,9%) e divulgação de conhecimento técnico e científico (93,3%). A exceção foi a teleconsultoria, definida como parcialmente implantada (74,4%). Entretanto, quando analisados isoladamente, observa-se que nem todos os indicadores e subcomponentes dessa dimensão apresentaram bons resultados.

Com relação à telerregulação, apenas o subcomponente “papel dos profissionais telerreguladores do projeto” foi implantado (100%), enquanto o “papel dos profissionais reguladores da central de regulação” foi parcialmente implantado (71,4%) e o “papel dos profissionais das UBS” foi incipiente (32,1%).

Na teleconsultoria, destaca-se o resultado do indicador “grau de adesão dos profissionais solicitantes às teleconsultorias”, avaliado pela maioria dos informantes como muito baixo, e do “solicitações reavaliadas após devolução para APS por consultoria (%)”, que alcançou 1%. Com relação ao componente “teleconsulta”, apesar de implantado, alguns informantes relataram baixa procura de usuários pelo serviço.

No componente “gestão do projeto”, destacam-se os indicadores “existência de workshop com os profissionais da rede”, “elaboração de relatórios técnicos e/ou de desempenho para acompanhamento dos indicadores do projeto” e “atualização mensal dos painéis de monitoramento compartilhados com o complexo regulador” - todos com pontuações abaixo do esperado. Mesma situação no componente “divulgação de conhecimento técnico e científico”, no qual o indicador “realização de divulgação e compartilhamento das experiências durante o período de execução do projeto” também apresentou baixa pontuação.

### Indicadores de resultado e sua relação com o grau de implantação

Quanto aos nove indicadores de resultado, três estavam dentro dos parâmetros de referência e demonstraram coerência com o grau de implantação obtido no componente “telerregulação”, especialmente em relação aos subcomponentes “papel dos profissionais telerreguladores do projeto” e “papel dos profissionais reguladores da central de regulação”, bem como no componente “teleconsulta”. Foram eles: “redução do tempo médio em dias de espera para autorização dos casos enviados às especialidades (%)”, que atingiu uma redução de 26%; “redução do tempo médio em dias de espera nos casos regulados em prioridade alta (%)”, com redução de 79%; e “nível de satisfação dos usuários atendidos nas teleconsultas”, com 90 *net promoter score* (NPS) (Quadro 2).

**Quadro 2:** Indicadores de resultado (efeito) do projeto Regula+ Brasil. Recife, Pernambuco, Brasil, 2022.

RESULTADOS INTERMEDIÁRIOS	INDICADOR	META	VALOR BASE	RESULTADO *	RESULTADO ALCANÇADO	*STATUS*
Aumento da qualidade dos encaminhamentos realizados pelos profissionais da APS	Solicitações aprovadas na primeira avaliação (%) - total e por especialidade	Aumento de duas vezes ou atingir 40%	8% (ortopedia: 0%; reumatologia: 0%; endocrinologia: 13%; cardiologia: 7%; neurologia: 6%; psiquiatria: 6%)	9% (ortopedia: 13%; reumatologia: 7%; endocrinologia: 8%; cardiologia: 9%; neurologia: 13%; psiquiatria **)	Aumento de 1,13%	Não alcançado
Qualificação dos casos regulados pela atenção especializada	Agendamentos de casos regulados prioritários (vermelho e amarelo) para atendimento especializado (%) - total e por especialidade ***	Incremento de 10% em relação ao ano anterior (2019)	48% (ortopedia: 64%; reumatologia: 76%; endocrinologia: 34%; cardiologia: 31%; neurologia: 88%; psiquiatria: 23%)	29% (ortopedia: 45%; reumatologia: 36%; endocrinologia: 13%; cardiologia: 25%; neurologia: 41%; psiquiatria **)	Redução de 19%	Não alcançado
Qualificação do processo de regulação assistencial das especialidades envolvidas no projeto	Redução do tempo médio em dias de espera para autorização dos casos enviados às especialidades (%) - total e por especialidade	Redução de 25% ou mais em relação ao ano anterior (2019)	119 dias (média) ortopedia: 323; reumatologia: 203; endocrinologia: 35; cardiologia: 127; neurologia: 218	88 dias (média) ortopedia: 244; reumatologia: 310; endocrinologia: 19; cardiologia: 105; neurologia: 145	Redução de 26% (média)	Alcançado
Redução do número de encaminhamentos desnecessários à atenção especializada	Redução de novos encaminhamentos nas filas de espera (%) - total e por especialidade	Redução de no mínimo 15%	2.637 novas solicitações/mês (ortopedia: 224; reumatologia: 386; endocrinologia: 599; cardiologia: 1.013; neurologia: 415; psiquiatria: 1.032)	4.431 novas solicitações no mês da mensuração ortopedia: 332; reumatologia: 503; endocrinologia: 1.764; cardiologia: 1.249; neurologia: 583; psiquiatria **)	Aumento de 68%	Não alcançado
Promoção do acesso equânime e em tempo oportuno	Redução do tempo médio em dias de espera nos casos regulados em prioridade alta (%) - total e por especialidade	Redução de no mínimo 25%	163 dias (ortopedia: 69; reumatologia: 3; endocrinologia: 6; cardiologia: 54; neurologia: 36; psiquiatria: 42)	35 dias (ortopedia: 20; reumatologia: 114; endocrinologia: 3; cardiologia: 26; neurologia: 14; psiquiatria **)	Redução de 79%	Alcançado
	Redução do tempo médio em dias de espera nos casos regulados em prioridade média e baixa (%) - total e por especialidade	Redução de no mínimo 25%	33 dias (ortopedia: 69; reumatologia: 39; endocrinologia: 6; cardiologia: 54; neurologia: 36; psiquiatria: 42)	76 dias (ortopedia: 196; reumatologia: 169; endocrinologia: 2; cardiologia: 54; neurologia: 17; psiquiatria **)	Aumento de 130%	Não alcançado
Redução de filas e tempo de espera para agendar atendimentos nas especialidades envolvidas no projeto	Redução no número de encaminhamentos em fila de espera (%) - total e por especialidade	Redução no mínimo de 40%	70.311 solicitações em fila (ortopedia: 10.091; reumatologia: 13.854; endocrinologia: 8.321; cardiologia: 24.872; neurologia: 13.173)	80.405 solicitações em fila (ortopedia: 11.019; reumatologia: 16.285; endocrinologia: 11.103; cardiologia: 27.445; neurologia: 14.553)	Aumento de 14%	Não alcançado
Melhor gestão das solicitações pendentes em filas de espera						
Maior suporte clínico às demandas assistenciais dos profissionais da APS	Nível de satisfação dos profissionais atendidos nas teleconsultorias	> 85 NPS	-	-	Não disponível apenas para Recife	-
Ampliação do conhecimento e do uso da telessaúde como resposta aos problemas na oferta de atenção especializada	Nível de satisfação dos usuários atendidos nas teleconsultas	> 85 NPS	-	-	90 NPS	Alcançado

APS: atenção primária à saúde; NPS: *net promoter score*.

Fonte: elaboração própria.

* Valor após 12 meses de projeto (maio/2020 a maio/2021);

** A especialidade de psiquiatria entrou no rol de especialidades do projeto em novembro de 2020, não apresentando dados acumulados de 12 meses necessários para o estudo;

*** Percentuais estimados com base no banco de dados extraído da API do Sistema Nacional de Regulação (SISREG).

Em contrapartida, cinco indicadores não alcançaram as metas preconizadas: “solicitações aprovadas na primeira avaliação (%)” (> 1,13%); “agendamento de casos regulados prioritários (vermelho e amarelo) para atendimento especializado (%)” (< 19%); “redução de novos encaminhamentos na fila de espera (%)” (> 68%); “redução do tempo médio em dias de espera nos casos regulados em prioridade média e baixa (%)” (> 130%); “redução no número de encaminhamentos em fila de espera (%)” (> 14%) - os quais demonstraram coerência com o grau de implantação obtido no componente “telerregulação”, subcomponente “papel dos profissionais das UBS”.

O indicador “nível de satisfação dos profissionais atendidos nas teleconsultorias” não pôde ser avaliado na pesquisa, visto que a equipe do projeto não tinha esse dado exclusivamente para os profissionais do município.

Assim, uma análise global entre o grau de implantação de estrutura e processo do projeto Regula+ Brasil e o desempenho dos indicadores de efeito parece demonstrar que a implantação não influenciou no alcance dos resultados. Contudo, quando considerados os componentes, subcomponentes e indicadores, isoladamente, sobretudo da dimensão processo, observa-se coerência entre eles.

O desempenho alcançado pelos indicadores “redução do tempo médio em dias de espera para autorização dos casos enviados às especialidades (%)” e “redução do tempo médio em dias de espera nos casos regulados em prioridade alta (%)” deve-se ao fato de estarem diretamente ligados às atividades desenvolvidas pelos profissionais telerreguladores do projeto e pelos reguladores da central de regulação do município.

Por outro lado, os indicadores “solicitações aprovadas na primeira avaliação (%)”, “agendamento de casos regulados prioritários para atendimento especializado (%)”, “redução de novos encaminhamentos na fila de espera (%)”, “redução do tempo médio em dias de espera nos casos regulados em prioridade média e baixa (%)” e “redução no número de encaminhamentos em fila de espera (%)” estariam sob maior influência da atuação dos profissionais das UBS, seja no acompanhamento e reenvio das solicitações devolvidas pelos profissionais telerreguladores, na redução e melhor qualificação dos encaminhamentos à atenção especializada ou no acolhimento das demandas sensíveis à APS por meio do serviço de teleconsultoria.

Outro ponto diz respeito à coerência entre o grau de implantação do subcomponente “papel dos profissionais das UBS” e do componente “teleconsultoria” e o desempenho inadequado do indicador “adesão profissionais da APS”, avaliado como muito baixo.

A atuação dos profissionais das UBS, principalmente no que diz respeito ao acompanhamento das solicitações devolvidas pela equipe do projeto, foi apontada pelos informantes-chave como um dos grandes entraves à obtenção de melhores resultados, especialmente pelo grande volume de solicitações devolvidas. Uma vez que tanto os profissionais telerreguladores do projeto quanto os reguladores da central de regulação dependiam do retorno das UBS para concluir o processo de regulação, a maior parte dessas solicitações ainda permaneciam retidas no sistema.

## Discussão

Os indicadores utilizados neste estudo para avaliar a influência do grau de implantação sobre os resultados do projeto Regula+ Brasil no Município de Recife forneceram informações importantes sobre a regulação assistencial. O grau de implantação total foi avaliado como implantado, assim como o grau de implantação das dimensões estrutura e processo, porém com diferentes níveis entre componentes e subcomponentes. O desempenho insatisfatório da maioria dos indicadores de efeito pareceu demonstrar que a implantação do projeto não influenciou no alcance dos resultados esperados. Entretanto, faz-se necessário analisar essas relações de forma mais minuciosa, confrontando o grau de implantação de componentes e subcomponentes com os efeitos.

Para a telerregulação, a avaliação a partir de cada subcomponente possibilitou, além de uma visão mais clara sobre sua implantação, identificar seu ponto de maior fragilidade. Observou-se que o subcomponente “papel dos profissionais das UBS” foi não apenas o único avaliado como não implantado, mas também parece ter sido o que repercutiu mais negativamente no alcance dos resultados.

Um dos principais fatores que limitaram sua implantação refere-se à incipiente rotina nas UBS de acompanhamento e reenvio das solicitações devolvidas pelos profissionais do projeto por ausência e/ou necessidade de complementação de dados clínicos. Dados levantados pela central de regulação de Recife ^18^ apontaram que cerca de 60% das solicitações pendentes nas filas de espera tiveram de ser devolvidas às UBS por falta e/ou necessidade de esclarecimentos clínicos. Além disso, apenas 17% haviam sido reenviadas pelas unidades com as informações solicitadas pelo projeto.

Essa realidade pode ter limitado a finalização da ação regulatória de muitos encaminhamentos, não gerando o impacto pretendido, principalmente no que diz respeito à redução das filas e do tempo de espera e à maior qualificação das solicitações.

A insuficiente transferência de informações clínicas, as falhas nos critérios de encaminhamento e as dificuldades no fluxo de informações entre a APS e a regulação são importantes obstáculos à regulação e ao seu papel de colaboradora no processo de coordenação do cuidado, podendo retardar ou mesmo impedir o acesso dos usuários aos serviços de saúde especializados ^19^^,^^20^^,^^21^.

Com relação ao componente “teleconsultoria” (serviço de suporte à regulação para garantir maior resolutividade da APS), o projeto utilizou um canal 0800, o qual os profissionais das UBS poderiam acionar para discussão de casos e/ou solicitações sinalizadas como sensíveis à APS pela equipe do projeto e se encontravam devolvidas no SISREG. Contudo, apesar da disponibilidade de estrutura e profissionais para o serviço, os resultados apresentados neste estudo identificaram baixa adesão dos profissionais das UBS, corroborando outros estudos ^22^^,^^23^^,^^24^^,^^25^^,^^26^.

Como exemplo de resultados alcançados pelo projeto em outras localidades, observaram-se redução de fila e de tempo de espera para consultas prioritárias em mais de 50% em Porto Alegre (Rio Grande do Sul) e Belo Horizonte (Minas Gerais), redução de 40% no número de novos encaminhamentos no Amazonas, além de aumento da qualificação dos encaminhamentos no Distrito Federal ^27^ - resultados não alcançados em Recife, com exceção do tempo de espera dos casos prioritários.

O componente “teleconsulta”, que não fazia parte do escopo inicial do projeto, foi incluído com o objetivo de reduzir o impacto da pandemia de COVID-19 no adiamento ou cancelamento de consultas eletivas e, no período de maio de 2020 a setembro de 2020, 622 teleconsultas foram realizadas ^28^. Em todo o mundo, a pandemia desencadeou a implantação de serviços de telemedicina para permitir a continuidade do cuidado aos pacientes com diferentes condições clínicas ^29^^,^^30^^,^^31^.

Os protocolos clínicos e de acesso utilizados pela equipe do projeto foram apontados pelos informantes como insuficientes, o que limitou a maior adesão e a condução do projeto. Os protocolos são ferramentas de gestão e de cuidado, pois tanto orientam as decisões dos profissionais solicitantes quanto atuam como referências que modulam a avaliação das solicitações pelos médicos reguladores ^32^. Contudo, para se tornarem efetivos, precisam ser articulados a processos que aumentem a capacidade clínica das equipes, que fortaleçam práticas de microrregulação nas UBS e que propiciem a comunicação entre unidades básicas, centrais de regulação e serviços especializados ^32^. Portanto, é necessária a adequação desses protocolos à realidade local da APS e à oferta assistencial do município.

Devido à pandemia, a “existência de workshop com os profissionais da rede” ocorreu quase seis meses depois do início das atividades do projeto. Apenas outro evento com os profissionais das UBS foi realizado, já na fase de encerramento, sendo apontado pelos informantes como fator que poderia ter auxiliado na maior divulgação e adesão dos profissionais. Esse achado corrobora outros estudos que citam aspectos organizacionais como fatores relevantes para adoção e incorporação da telessaúde ^24^^,^^33^^,^^34^^,^^35^^,^^36^^,^^37^^,^^38^^,^^39^^,^^40^.

A telemedicina desencadeia uma série de alterações nas formas de coordenação, processos de trabalho e relações de poder. Moehr et al. ^37^ apontaram que a introdução da telessaúde no Canadá, sem planejamento, tempo de preparo e estabelecimento de rotinas, foi um dos fatores de impedimento ao seu uso. Para Jennett et al. ^36^, fatores organizacionais têm sido indicados como responsáveis por até 30% das falhas na adoção de inovação tecnológica.

Estudo que analisou estratégias no processo de implementação de uma solução digital observou elevados níveis de coerência e entusiasmo dos participantes após a organização de workshops, assim como a importância de suporte durante a implementação, com a presença de pessoas que liderem o processo, que promovam a utilização da telessaúde e que motivem os participantes ^41^.

Tanto os relatórios e painéis de acompanhamento dos indicadores quanto o canal de comunicação entre a equipe do projeto e as UBS foram apontados como ferramentas que poderiam ter sido melhor aproveitadas. A falta de cultura e capacidade organizacional para coletar, manejar e avaliar os dados em saúde inviabilizou os enormes ganhos que mecanismos de feedback podem produzir a ações complexas ^42^^,^^43^. Entender as barreiras e virtudes da oferta dessas soluções é fundamental para melhorar esses resultados ^44^^,^^45^^,^^46^.

Além disso, envolver os diversos atores na escolha da solução é igualmente uma tarefa importante. Segundo Bradford et al. ^46^, muitas vezes ignora-se a capacidade dos atores envolvidos na intervenção de identificar as necessidades e propor soluções efetivas. Nesse contexto, é fundamental que os serviços sejam não apenas centrados nas demandas dos usuários (como premissa fundamental), mas também nas necessidades dos profissionais de saúde, pois são eles que frequentemente dirigem as demandas dos pacientes nos sistemas de telessaúde.

Quanto à divulgação de conhecimento técnico e científico, mesmo com publicações sobre o tema ^16^^,^^28^^,^^47^, alguns informantes apontaram para a importância de maior divulgação e compartilhamento das experiências, principalmente em relação aos profissionais das UBS, o que poderia ter contribuído para maior adesão. A efetividade de um projeto de telessaúde envolve um conjunto de aspectos, sobretudo no que diz respeito ao envolvimento dos *stakeholders*^42^.

Diante dos achados, compreende-se o comportamento dos profissionais de saúde como elemento-chave para implantação de intervenções efetivas ^48^, porém não é possível analisar essa conduta de maneira desassociada do componente político. O projeto Regula+ Brasil foi implementado a partir do PROADI-SUS, programa que conta com o envolvimento de diversas instâncias tomadoras de decisão, que vão desde o Ministério da Saúde e o hospital de excelência até o nível local, as quais figuram como forças influenciadoras das decisões: atores sociais, poder e políticas, instituições, interesses e ideias ^49^. Trata-se, portanto, de um fenômeno complexo, constituído e alimentado pelo diverso em interação. De igual modo, as estratégias para lidar com essa realidade também necessitam ser complexas ^50^.

Como limitação deste estudo, devido à escassez de indicadores e parâmetros de avaliação nos documentos oficiais do projeto, principalmente para a dimensão processo, tais valores de referência foram empiricamente derivados. No entanto, ao submeter o modelo lógico e a matriz de julgamento a um painel de especialistas inseridos em diferentes espaços de envolvimento do projeto e ao confrontar fontes, buscou-se ampliar a validade dos instrumentos.

## Conclusões

Sabe-se que as dificuldades operacionais e as fragilidades dos mecanismos de coordenação do cuidado nas redes de atenção à saúde têm apontado para a necessidade de adoção de novos arranjos para garantir um acesso mais equânime e integral aos usuários, fato que tem estimulado na última década a incorporação de tecnologias, principalmente a partir da pandemia de COVID-19. No entanto, apesar das vantagens no uso dessas tecnologias no processo regulatório, ainda parecem existir barreiras à sua devida implantação e, especialmente, à obtenção de resultados efetivos.

Os resultados deste estudo sugerem que deve haver certa cautela com a utilização de tecnologias de apoio à comunicação e à informação, sob pena de se configurar nova burocratização e novo obstáculo de acesso, quando o que se precisa é uma regulação viva e centrada nas necessidades dos usuários mais do que em normas ou procedimentos ^51^. A incorporação da telessaúde exige reorganização das equipes para adequação dos processos de trabalho, rearranjo das agendas de profissionais e sua inclusão como prática de educação permanente, que permita o aprimoramento profissional e o acesso ao apoio necessário ^52^^,^^53^. Ainda, trata-se de um processo contínuo de construção e avaliação.

Nesse sentido, é necessário fortalecer as equipes de regulação no que diz respeito ao quantitativo e à capacidade técnica dos operadores do SISREG nas UBS para administração das demandas. Além disso, a adequação dos protocolos clínicos e de encaminhamento à realidade assistencial do município é fundamental, levando em conta a participação conjunta de gestores e profissionais da regulação e atenção básica.

Este estudo também apontou para a importância de se estabelecer, desde o início da implantação do projeto, um processo de educação continuada para maior envolvimento dos profissionais das UBS, tanto dos profissionais solicitantes quanto dos operadores do SISREG - seja por meio de capacitações e/ou reuniões periódicas com a equipe, abordando objetivos e operacionalização do projeto, divulgação dos instrumentos (protocolos) e a importância da atuação dos diferentes atores (responsabilização compartilhada), seja com a apresentação dos resultados parciais e das dificuldades observadas. Estabelecer um canal de comunicação direto e permanente com os profissionais da APS, com a escolha de atores-chave (coordenação da atenção básica municipal e coordenação da equipe médica, por exemplo), para possibilitar maior articulação entre as ações da equipe do projeto e das equipes de saúde da família pode ser uma medida pertinente. Ademais, é importante a atuação permanente da central de regulação junto aos operadores do SISREG para continuidade do acompanhamento das demandas, identificação de gargalos e aprimoramento dos processos de trabalho.
